# The relationship between hypoalbuminaemia, tumour volume and the systemic inflammatory response in patients with colorectal liver metastases

**DOI:** 10.1038/sj.bjc.6601886

**Published:** 2004-06-22

**Authors:** R Al-Shaiba, D C McMillan, W J Angerson, E Leen, C S McArdle, P Horgan

**Affiliations:** 1 University Department of Surgery, Royal Infirmary, Glasgow G31 2ER, UK; 2 Department of Radiology, Royal Infirmary, Glasgow G31 2ER, UK

**Keywords:** colorectal liver metastases, hypoalbuminaemia, tumour volume, C-reactive protein, liver enzymes

## Abstract

The relationship between hypoalbuminaemia, tumour volume and C-reactive protein was examined in patients with colorectal liver metastases (*n*=57). On multiple regression analysis, albumin concentrations were independently associated with C-reactive protein (*r*=0.56, *P*<0.001) but not percentage hepatic replacement (*P*=0.34). These results show that hypoalbuminaemia is associated with the presence of a systemic inflammatory response rather than tumour volume in patients with colorectal liver metastases.

It has long been recognised that, in patients with advanced cancer, the presence of hypoalbuminaemia is associated with poor outcome (Franch-Arcas, 2001). In the past, this hypoalbuminaemia has been thought to be the result of nutritional depletion secondary to the tumour. Recently, however, it has been postulated that the reduction in albumin concentration is secondary to the presence of a systemic inflammatory response, as evidenced by elevated circulating concentrations of C-reactive protein (McMillan *et al*, 2001).

The nature of the relationship between circulating albumin concentration, tumour burden and the systemic inflammatory response has not been established. However, if the presence of a systemic inflammatory response rather than tumour burden were to determine the development of the hypoalbuminaemia associated with advanced cancer, then it might be expected that there would be a stronger relationship between the presence of hypoalbuminaemia and the systemic inflammatory response compared with that between hypoalbuminaemia and tumour volume.

The aim of the present study was to examine the relationship between circulating albumin concentration, the tumour burden and the systemic inflammatory response in patients with colorectal liver metastases.

## MATERIAL AND METHODS

### Patients

Patients who had undergone resection of a primary colorectal cancer, who then developed liver metastases and who were referred for assessment for liver resection, were considered eligible for study. As part of their assessment, patients underwent dual phase spiral-computed tomography of the liver. In addition a blood sample was taken for measurement of routine liver enzymes, albumin and C-reactive protein. Only those patients who had no evidence of extrahepatic disease were included in the study. None of the patients had recent chemotherapy.

The study was approved by the Research Ethics Committee of Glasgow Royal Infirmary.

### Biochemical profile

Albumin, C-reactive protein, alkaline phosphatase, aspartate transaminase, alanine transaminase and bilirubin concentrations were assayed using standard methods. Intra- and inter-assay coefficients of variation were better than 5 and 10% respectively.

### Tumour volume and PHR

Tumour volume and percentage hepatic replacement (PHR) were measured using a similar approach to that previously described (Purkiss and Williams, 1992; Dworkin *et al*, 1995). Contrast enhanced CT-scans were performed using a Somatom plus 4 spiral-CT scanner (Siemens AG, Erlangen, Germany).

The cross-sectional areas of all the metastatic lesions in each 5 mm slice were measured by mapping the perimeter of the region of interest. The total tumour volume was calculated as the sum of the tumour areas in all slices multiplied by the slice thickness. The total liver volume was measured in a similar manner, the PHR calculated as the ratio of tumour to total liver volume multiplied by 100.

To assess the reproducibility of the method used, two observers independently measured tumour and liver volumes and PHR in 10 randomly selected patients from the above cohort. The intra-class correlation coefficients were 0.97 or greater for all comparisons.

To assess the accuracy of the CT-derived measurements of tumour volume, the CT measurements of tumour volume were compared with the weight of freshly resected tumour in a separate cohort of nine patients undergoing liver resection. The correlation coefficient between the CT-derived measurements of tumour volume and the weight of the resected tumour was greater than 0.99 (*P*<0.001).

### Statistics

The relationships between albumin, PHR and C-reactive protein were analysed using Pearson correlation coefficients and multiple linear regression analysis with albumin as the dependent variable. PHR and C-reactive protein concentration were logarithmically transformed to normalise their distributions. The appropriateness of the regression model was confirmed by examining residuals graphically. Associations involving liver enzymes and bilirubin were analysed using Spearman's rank correlation coefficient. Comparisons between groups of patients were carried out Fisher's exact test or the Mann-Whitney *U*-test as appropriate. SPSS for Windows (SPSS Inc., Chicago, IL, USA) was used for analysis.

## RESULTS

The characteristics of the patients studied are shown in Table 1

**Table 1:** Characteristics and biochemical parameters of patients with colorectal liver metastases in the presence and absence of hypoalbuminaemia

	**All patients (*n*=57)**	**Albumin ⩾35 g l^−1^ (*n*=36)**	**Albumin <35 g l^−1^ (*n*=21)**	*P*-value*
Age (years)	66 (46–88)	66 (46–83)	69 (46–88)	0.408
Sex male/female	38/19	25/11	13/8	0.560
				
*Liver metastases*				
Single/multiple	14/43	11/25	3/18	0.169
Unilobar/bilobar	32/25	24/12	8/13	0.068
Tumour volume (cm^3^)	126 (3–2006)	78 (3–1816)	248 (10–2006)	0.175
PHR (%)	7 (<1–58)	4 (<1–46)	15 (<1–58)	0.186
				
*Biochemistry*				
Albumin (g l^−1^)	37 (18–48)	40 (35–48)	31 (18–34)	
Alkaline phosphatase (U l^−1^)	253 (83–1750)	255 (115–1750)	250 (83–1530)	0.810
Aspartate transaminase (U l^−1^)	29 (13–770)	25 (14–72)	48 (13–770)	0.055
Alanine transaminase (U l^−1^)	19 (9–380)	16 (10–110)	31 (9–380)	0.090
Bilirubin (*μ*mol l^−1^)	14 (7–240)	13 (7–22)	17 (7–240)	0.034
C-reactive protein (mg l^−1^)	38 (<6–247)	29 (<6–161)	82 (6–247)	0.006

Median (range),

* comparison of groups with albumin ⩾35 g l^−1^ and albumin <35 g l^−1^.

. The majority were male, were over the age of 60 years, and had multiple liver metastases, a PHR less than 25%, an albumin concentration in the normal range and an elevated C-reactive protein concentration. Patients with hypoalbuminaemia had significantly higher concentrations of bilirubin (*P*=0.034) and C-reactive protein (*P*=0.006).

There were significant correlations between albumin and PHR (*r*=−0.34, *P*=0.010), albumin and C-reactive protein (*r*=−0.56, *P*<0.001) and PHR and C-reactive protein (*r*=0.43, *P*=0.001). A multiple regression model combining PHR and C-reactive protein as predictor variables (*r*=−0.57, *P*<0.001) did not significantly improve on a model based on C-reactive protein alone (Table 2

**Table 2:** Analysis of variance for regression of albumin on C-reactive protein (CRP) and percentage hepatic replacement by tumour (PHR)

**Source of variation**	**Degrees of freedom**	**Sum of squares**	**Mean square**	**F**	***P*-value**
Total	56	2146.035			
Regression	2	707.824	353.912	13.288	<0.001
CRP alone	1	683.266	683.266	25.691	<0.001
PHR adjusted for CRP	1	24.558	24.558	0.922	0.34
PHR alone	1	245.799	245.799	7.114	0.01
CRP adjusted for PHR	1	462.025	462.025	17.347	<0.001
Residual	54	1438.212	26.634		

). In the combined model, C-reactive protein was a significant independent predictor of albumin concentration adjusted for PHR (*P*<0.001), while the relationship between albumin and PHR adjusted for C-reactive protein was not significant (*P*=0.34). The relationship between circulating concentrations of albumin and C-reactive protein is shown in Figure 1

**Figure 1:**
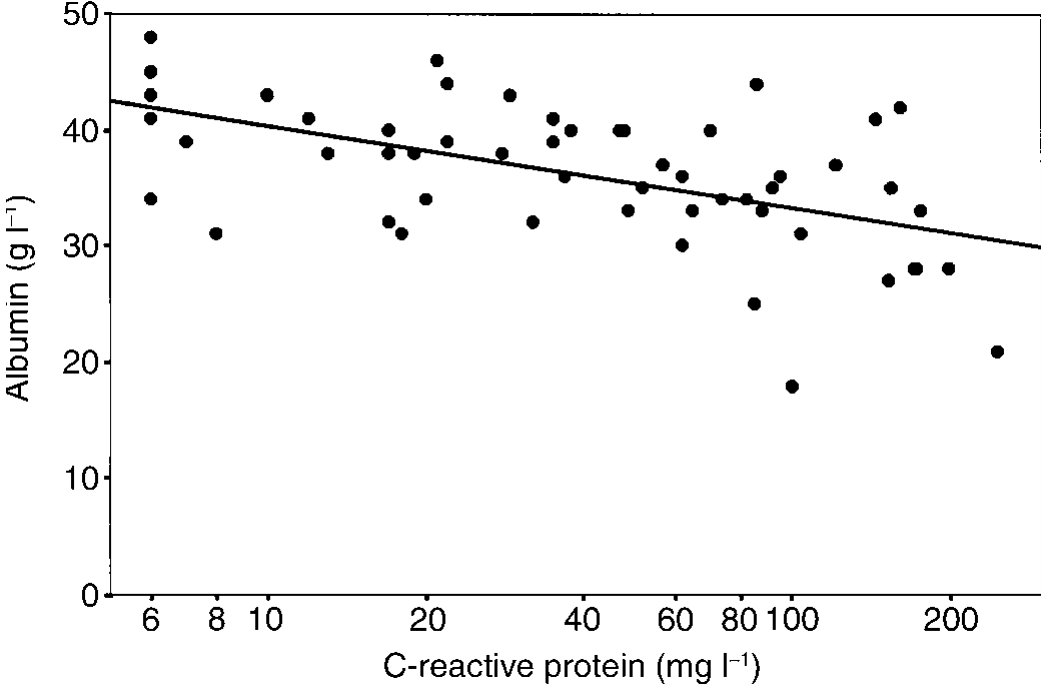
The relationship between albumin and C-reactive protein concentrations in patients with colorectal liver metastases (*n*=57).

.

There were significant rank correlations between PHR and alkaline phosphatase (*r*_s_=0.61, *P*<0.001), aspartate transaminase (*r*_s_=0.48, *P*=0.001), alanine transaminase (*r*_s_=0.32, *P*=0.025) and bilirubin (*r*_s_=0.44, *P*=0.002). Of these liver enzymes and bilirubin, only aspartate transaminase was significantly associated with C-reactive protein (*r*_s_=0.29, *P*=0.045) and albumin (*r*_s_=−0.34, *P*=0.016).

## DISCUSSION

It has been recognised for some time that the degree of biochemical upset in patients with advanced cancer depends not only on the tumour burden but also the site of the disease. In advanced colorectal cancer where the tumour is disseminated only to the liver, tumour size is readily assessed by computed tomography and therefore provides a model in which to examine the relationship between hypoalbuminaemia and tumour volume.

It is well recognised that albumin concentration is a stage independent prognostic factor in patients with advanced colorectal cancer (O'Gorman *et al*, 2000; Dixon *et al*, 2003). In the present study, there was only a weak relationship between albumin concentrations and tumour volume, and therefore the prognostic value of hypoalbuminaemia would not appear to be primarily dependent on tumour burden.

In contrast, albumin was strongly associated with the magnitude of the systemic inflammatory response as evidenced by circulating concentrations of C-reactive protein. Furthermore, in a model combining albumin, C-reactive protein and PHR, there was no relationship between albumin and PHR when adjusted for the effect of C-reactive protein. Therefore, the results of the present study are consistent with the concept that the systemic inflammatory response is a major determinant of albumin concentrations in patients with advanced colorectal cancer.

It may be that the presence of a chronic systemic inflammatory response, with its increased demand for specific amino acids for acute phase protein synthesis, promotes the degradation of available body protein including albumin (Preston *et al*, 1998; McMillan *et al*, 2001). Indeed, this relationship between nutritional decline, albumin concentrations and the systemic inflammatory response has recently been exploited to form a new prognostic score in patients with advanced non-small-cell lung cancer (Forrest *et al*, 2003).

The results of the present study also showed that increased activity of the enzymes alkaline phosphatase, aspartate transaminase and alanine transaminase reflect increased tumour burden. In particular, raised alkaline phosphatase activity, a recognised prognostic factor, was significantly correlated with tumour volume and therefore its prognostic value would appear to be related to the size of the liver metastases. These increased enzyme activities are consistent with previous work in patients with colorectal liver metastastases (Rougier *et al*, 1995)

In summary, the results of the present study suggest that, in patients with advanced colorectal cancer, hypoalbuminaemia is secondary to the presence of a systemic inflammatory response rather than nutritional depletion by the tumour.
